# NetMets: software for quantifying and visualizing errors in biological network segmentation

**DOI:** 10.1186/1471-2105-13-S8-S7

**Published:** 2012-05-18

**Authors:** David Mayerich, Chris Bjornsson, Jonathan Taylor, Badrinath Roysam

**Affiliations:** 1 Beckman Institute for Advanced Science and Technology, University of Illinois at Urbana-Champaign, USA; 2 Center for Biotechnology and Interdisciplinary Studies, Rensselaer Polytechnic Institute, New York, USA; 3 Department of Electrical and Computer Engineering, University of Houston, Texas, USA

## Abstract

One of the major goals in biomedical image processing is accurate segmentation of networks embedded in volumetric data sets. Biological networks are composed of a meshwork of thin filaments that span large volumes of tissue. Examples of these structures include neurons and microvasculature, which can take the form of both hierarchical trees and fully connected networks, depending on the imaging modality and resolution. Network function depends on both the geometric structure and connectivity. Therefore, there is considerable demand for algorithms that segment biological networks embedded in three-dimensional data. While a large number of tracking and segmentation algorithms have been published, most of these do not generalize well across data sets. One of the major reasons for the lack of general-purpose algorithms is the limited availability of metrics that can be used to quantitatively compare their effectiveness against a pre-constructed ground-truth. In this paper, we propose a robust metric for measuring and visualizing the differences between network models. Our algorithm takes into account both geometry and connectivity to measure network similarity. These metrics are then mapped back onto an explicit model for visualization.

## Introduction

Three-dimensional biomedical data sets often contain complex anatomical structures that are difficult to segment and reconstruct. Of particular interest are filament networks embedded in volumetric data. Examples of these include vascular and neuronal networks. With increased use of high-throughput imaging, there has been significant interest in fast and accurate segmentation algorithms for large data sets. However, segmentation of filament networks in microscopy data sets continues to be a difficult problem. While there has been an effort to distribute tracking algorithms both commercially and as open source through software packages such as the Farsight Toolkit http://www.farsight-toolkit.org, most algorithms are optimized for specific data sets and imaging modalities. In fact, funding initiatives like the DIADEM Challenge [1] have been designed to motivate researchers to create generalized segmentation algorithms that work across several data sets.

**Figure 1:**
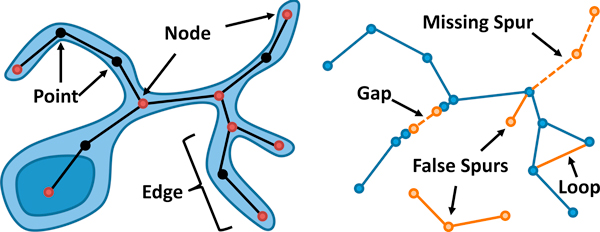
**Explicit representation of a neuron model**. (left) The network can be represented as a graph structure, where nodes are end points and branch points. Each fiber is represented by a single edge. (right) The same network is shown with several common errors introduced.

One of the major roadblocks preventing broad use of these algorithms is the inability to compare the effectiveness of filament segmentation results, which can contain multiple geometric and connectivity errors (Figure 1). In fact, the only metric that we are aware of for comparing segmentation results was created to help evaluate submissions to the DIADEM Challenge. In this paper, we propose efficient and robust metrics for comparing complex interconnected structures. We show that these metrics can also be used to visualize differences in networks to better qualify the benefits and limitations of segmentation approaches.

### Previous work

There is an extensive body of published work on filament segmentation in biomedical data sets. A review of vessel extraction techniques for vascular trees is given by Kirbas and Quek [2] and a review of neuronal tracing methods is available by Donohue and Ascoli [3]. Semi-automated techniques are often used in commercial software and recent methods have been proposed to significantly speed up skeletonization based on user input [4]. Thinning algorithms are often used for segmentation of high-contrast images [5-7]. While these generally rely on local or global thresholds, recent methods have been proposed that are threshold independent [8] and more robust to surface perturbations [9]. Filament tracking techniques have been developed for low-contrast data, such as neurons in confocal data sets, and rely on the intensity gradient across the filament cross-section [10-12]. Several techniques have also been proposed for making segmentation more robust. These include multi-hypothesis tracking [13] and post-processing [14,15].

While new and improved segmentation techniques are proposed yearly, there are few methods for quantitatively comparing results to an established ground truth. The DIADEM metric [16,17] and Path2Path [18] are the only quantitative technique that we are aware of specifically designed to compare neurons. However, we also consider other metrics and measurements that could be applied to this problem. In the following sections, we describe some alternatives for evaluating differences in explicit networks. We place a particular focus on metrics previously developed for validation in neuronal and vascular tree segmentation. These potential metrics are organized into two groups: geometric methods and topological methods.

### Geometric methods

The most basic approach for comparing an explicit representation is the use of standard geometry metrics, such as those used for validation in surface segmentation and surface simplification (level-of-detail). An overview of these methods as they are applied to geometric models is provided by Luebke [19]. While these metrics are designed to evaluate two-dimensional surfaces, applying them to interconnected networks of one-dimensional centerlines is straightforward. Two of the most common examples of these geometric techniques are the mean squared error (MSE) and the Housdorff distance. In addition, the Path2Path algorithm [18] was recently proposed as a geometric approach specifically designed for comparing similarity between neurons.

We first consider the mean squared error, which is computed by averaging the square of minimum distances from one geometric model *A *to another geometric model *B*. In the ideal case *A *= *B *and therefore *MSE*(*A, B*) = 0. One disadvantage of this measurement is that it is not commutative, meaning that generally *MSE*(*A, B*) ≠ *MSE*(*B, A*). The Hausdorff distance addresses this issue by computing a mutual metric that is the maximum of minimum distances between *A *and *B*. This metric is commutative, however the metric weighting is based solely on the largest distance between *A *and *B*. This can easily result in ignoring more relevant errors that occur near the ground truth. This also makes the Hausdorff distance, and to a lesser degree the MSE, sensitive to spurious geometric components that may appear some distance from the ground truth model. Finally, both of these metrics are insensitive to errors in connectivity, which can exist independently to the network shape.

The Path2Path metric was proposed as a method for comparing the geometric characteristics of a neuron in order to facilitate queries into online neuronal databases. This metric forgoes the standard graph representation of a neuronal tree in favor of a collection of geometric paths that extend from the root node to the end of each neuronal process.

The neurons are then compared by determining the amount of energy required to optimally morph the set of paths in *A *to match the set of paths in *B*. Since the connectivity is discarded, Path2Path is essentially a geometric measure. However, since many paths in the resulting set overlap, errors are difficult to spatially localize.

The geometry metric proposed in this paper is based on a similar principle to the MSE. The lack of commutativity is addressed by computing a bi-directional measurement, which is incorporated into the geometric FPR and FNR. The distance sensitivity is eliminated by scaling the error between *A *and *B *by an inverse non-linear Gaussian function that approaches unity at a relatively short distance from the model. In addition, this allows for errors to be spatially localized and the resulting metric can be visualized within a narrow constant range of 0[1].

### Topological methods

Recent methods proposed for validating tree-like segmentations of vasculature and neurons are based on topological approaches. These methods leverage the hierarchical structure of the model in order to quantify segmentation error. Unlike geometric approaches, these methods account for connectivity and can also incorporate some basic geometric information. The two methods that we address are the constrained Tree Edit Distance (TED) [20] and the DIADEM Metric [16].

The constrained TED provides a metric that identifies the number of edits that must be performed on a given model *A *in order for it to topologically match a second model *B*. These edits take the form of node insertions and deletions. This method has been proposed as a technique for quantifying the difference between two neuronal models [21]. However, the TED is not geometrically specific, since the metric depends only on branch position within the hierarchy relative to a root node and is independent of the spatial position and shape of structures in the tree.

The DIADEM Metric incorporates geometric characteristics of the two models in order to better map branches in the test case model to corresponding geometry in the ground truth. Branch points and end points, for example, are mapped between the models *A *and *B *using a proximity query. This constraint makes the topological analysis significantly more efficient while allowing a direct mapping between fibers (edges) in *A *and *B*. In addition, the path length for each fiber can then be directly compared in order to modify the metric based on geometric deviations of the fibers from the ground truth.

**Figure 2:**
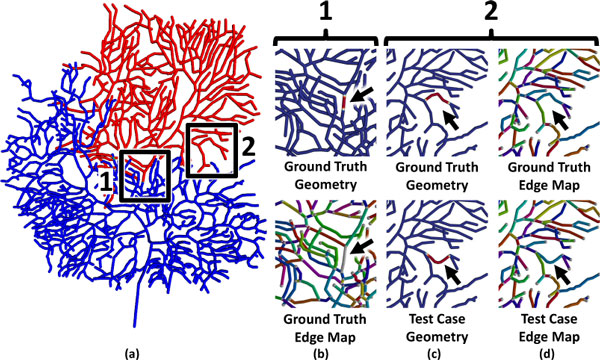
**Artifacts encountered when using hierarchical techniques**. (a) A purkinje cell from the Virtual Neuromorphology Electronic Database is shown with two errors introduced: (1) a gap in a fiber and (2) a geometric distortion. The red region indicates the error evaluated using a hierarchical metric. (b) Our proposed method correctly identifies the gap as a small geometric error corresponding with a single missing connection. (c) Our method can also identify a geometric distortion, even though there is no resulting error in topology. (d) Despite this error, edge mapping is consistent across the test case and ground truth.

One of the fundamental problems with current topological approaches is that they depend on the topology of the input models to be tree-like. Therefore, these methods cannot be directly applied to interconnected networks, such as microvascular networks and large-scale reconstructions of neural networks. In addition, current topological techniques are highly sensitive to errors in connectivity, which are commonly encountered using automated segmentation techniques. This is demonstrated in Figure 2, where a single break in connectivity results in penalizing a large portion of the tree that is otherwise accurately segmented.

The connectivity metric that we propose relies on mapping of branch points and end points between the ground truth and test case, which is similar to the initial approach taken by the DIADEM metric. We then use a graph traversal method to evaluate connectivity locally [22], which allows our algorithm to compare non-hierarchical and interconnected networks. By removing the dependence on a hierarchical network model, our algorithm is also robust to connectivity errors that include gaps in the network. In addition, the NetMets software allows the error to be localized and visualized on a rendered image of the ground truth model (Figure 2b). Finally, we demonstrate that our algorithm can detect the deformation of a single fiber in the test case (Figure 2c). Since the topology of the network is still correct, this error is undetected using the constrained TED. Since the fiber length is maintained, this geometric error is also undetectible using the DIADEM metric. These errors are easily located and visualized by identifying mapped edges with a high geometric error in NetMets.

### Proposed methods

The method that we propose compares both the geometry and connectivity of two interconnected networks. Based on a single parameter σ, defining the sensitivity of the metric, our algorithm returns four normalized values characterizing the degree of similarity between two input networks. These metrics are then mapped onto the original input models so that differences between the networks can be visualized. In all cases presented here, the parameter σ is set to the mean fiber radius, however other values can be used. Higher values of σ result in a decrease in the detected error (FNR and FPR).

In the following sections, we describe the input to our proposed algorithm and define the terms used to process network models.

### Input models

The most common format for storing traced neurons is the SWC file. SWC files are supported by popular network simulation programs, including NEURON [23] and NETMORPH [24]. In addition, SWC is the most common format found in online neuron libraries used for connectomics research, including visualization [25] and simulation [26,27]. An SWC file represents neurons as a sequence of 3D points, along with their parent point. All points extend from the root node of the neuron, which is generally the cell body. While the SWC file is sufficient for representing trees, closed loops cannot be formed. Our algorithm supports tree-like structures loaded using the SWC format as well as tree-like and closed-loop models represented using the Alias/Wavefront OBJ file format.

### Terminology

When describing connectivity operations, we use the following terminology:

• A *node *is a junction where multiple fibers connect, or where a single fiber terminates.

• An *edge *is a filament that links two nodes.

• A *point *is a three-dimensional position that lies on the network skeleton.

Note that a fiber can consist of multiple points that describe its geometric shape. While these points are used to evaluate the network geometry, a fiber is represented topologically by a single edge (Figure 1). In addition, we recognize two primary connectivity errors:

• Gaps in fibers.

• Excess edges forming loops or spurs.

Note that a spur connected to correctly segmented geometry is identified as an error even though it is not strictly a topological change.

### Overview

The metric proposed in this paper provides false positive and false negative rates for network geometry and connectivity. The proposed geometry metric integrates a weighted distance function along all curves in a network. We show that this can be evaluated efficiently in $O\left(\frac{L}{\sigma }\text{log}\frac{L}{\sigma }\right)$ time, where *L *is the length of all fibers in the network and σ is a sensitivity parameter.

We measure connectivity differences by using geometric information to map between nodes and edges in both networks. We then find a set of edges and nodes common to the ground truth and test case. Excess features are then used to quantify the connectivity differences between the two networks.

### Geometry

In this section, we first identify the fundamental problem with applying the standard geometry metrics described previously to network segmentations. We then describe the method used by NetMets to address these issues. Error metrics such as MSE and the Hausdorff distance provide a global measure of model similarity, which is ideal when constructing a mesh based on a source model. However, these techniques are not robust for fibrous models, and often apply excessive penalties for relatively small errors. Consider a test case *T *that perfectly matches the ground truth *GT *for a neuron, except for a small length of fiber some distance *ε *from the cell. When measuring the mean L1-distance from *T *to *GT*, the networks will appear identical. However, computing the Hausdorff distance will result in a value of ≈ *ε*. Measuring the mean *L*1-distance from GT to T also provides a result of ≈ *ε *where *L *is the length of the spurious segment. In both cases, changing the distance *ε *significantly affects the error, even though the distance of the spurious fiber is likely irrelevant to improving the segmentation algorithm used. A more intuitive metric would scale some constant value by the length *L *of the detected segment. However, global application of a distance threshold minimizes the impact of errors that occur close to the network, such as oscillations and gaps in fibers. Given two networks *N*_1 _and *N*_2_, our proposed algorithm estimates the ratio of the length of fiber in *N*_1 _that has no correspondence in *N*_2 _to the total fiber length in *N*_1_. This estimate is computed by placing an implicit Gaussian envelope around *N*_2 _and integrating along the set of curves representing fibers in *N*_1_. In order to quantify both missed fibers and false positives, we perform a bi-directional measurement, comparing *N*_1 _to *N*_2 _as well as comparing *N*_2 _to *N*_1_.

In the following sections, we describe common geometric errors encountered in network segmentation. We then describe the theory behind our proposed measurement as well as implementation details and methods for improving accuracy.

### Common geometry errors

Errors in segmentation consist of both undetected and spurious fibers as well as deformations in fibers. Thinning algorithms [5] are sensitive to variations in the fiber surface, resulting in spurs that are not present in the ground-truth. This becomes more prominent when high-frequency surface features are present, such as dendritic spines in images of neurons. Since many segmentation algorithms require thresholding as a pre-processing step, noise and other artifacts can create spurious loops or gaps in the geometry.

Tracking methods [10,12] are more robust to many of these errors, however they rely on seed points for fiber detection. Incorrect placement of seed points can cause entire fibers to be missed. In addition, tracking algorithms are more stable, often causing false fibers to be significantly longer. This makes them more difficult to locate and remove using post-processing. Finally, variations in the image can cause segmented fibers to oscillate and deviate from the corresponding fibers in the ground truth.

### Geometry metric

Given two networks *N*_1 _and *N*_2_, our proposed metric returns a value that estimates the ratio between the length of fibers in *N*_1 _that do not exist in *N*_2 _and the total length of fibers in *N*_1_. This is described by the following equation:

(1) $$M\left(N_{1},N_{2}\right)=\frac{\int \left(N_{1}-N_{1}\cap N_{2}\right)}{\int N_{1}}$$

where *N*_1_∩*N*_2 _is the set of fibers common to both networks and integration refers to the length of fiber in the corresponding set. Since this metric does not consider the topological structure of each network, it may be helpful to think of *N*_1 _and *N*_2 _as the sets of all points that lie on the curves representing the corresponding network. However, it is impractical to evaluate the statement *N*_1_∩*N*_2 _for an explicit model since it is extremely unlikely that fibers in *N*_1 _and *N*_2 _will precisely overlap.

Consider the descretization of Equation 1 onto a three-dimensional grid with a voxel size of some small number *ε*. In this case, the explicit representation can be thought of as a set of points rasterized onto the underlying grid. The metric is then represented using:

(2) $$M\left(N_{1},N_{2}\right)=\frac{1}{n}\sum \left(N_{1}-N_{1}\cap N_{2}\right)$$

where *n *is the number of grid points in *N*1. While a small value of *ε *allows M to capture fine-scale differences between *N*_1 _and *N*_2_, the metric itself becomes unrealistically strict. We overcome this problem by scaling the points in *N*_1 _by a weighted distance field based on the geometry of *N*_2_:

(3) $$M\left(N_{1},N_{2}\right)=\frac{1}{n}\overset{n}{\underset{x\in N_{1}}{\sum }}\left(1-e^{\frac{d{\left(x,N_{2}\right)}^{2}}{2\sigma^{2}}}\right)$$

where *d*(*x, N*_2_) is the distance between *x *and the closest point in *N*_2 _and *σ *is a sensitivity parameter. Intuitively, this is the equivalent of placing a Gaussian envelope around *N*_2 _and weighting the points in *N*_1 _based on their position within this field. The value *σ *is the standard deviation of this Gaussian envelope.

An analysis that takes into account both spurious and undetected fibers requires a bidirectional measurement. Since the described metric *M*(*N*_1_, *N*_2_) provides an estimate of the fraction of *N*_1 _that is not contained in *N*_2_, a bidirectional measurement is used to determine the rate of false positives and false negatives:

(4) $$G_{FNR}=M\left(N_{GT},N_{T}\right)$$

(5) $$G_{FPR}=M\left(N_{T},N_{GT}\right)$$

where *G_FNR _*is the false negative rate, *G_FPR _*is the false positive rate, and *N_GT _*and *N_T _*are the ground-truth and test-cases respectively.

Note that Equation 3 can be determined for subsets of the network. The metric value for a single point is used for visualization while integration along a single fiber is used to determine weights for the connectivity metric.

### Implementation

*M *is evaluated by determining a set of points that lie on each network and resolving the distance function using a nearest-neighbor search. The explicit models are re-sampled at intervals of at most *εσ*, where *ε *is a parameter describing the degree of accuracy of the geometry measurement. Resampling is performed using linear interpolation, since this is the standard for representing neuronal models. However higher-order interpolants can be used by resampling the network model as a preprocessing step.

The distance function *d*(*x, N*_2_) is evaluated using a nearest-neighbor search. The sample points for *N*_2 _are stored in a kd-tree [28,29] and successive queries for all *x *∈ *N*_1 _are used to determine the distance to the closest point on *N*_2_. This distance is then used to evaluate the geometry metric (Equation 3).

### Accuracy

The accuracy of the geometry metric is of particular interest since the distance function *d*(*x, N*) (Equation 3) is determined using a nearest-neighbor search, and therefore dependent on the spacing between sample points. In addition, the distance field is scaled by a nonlinear Gaussian function. While this reduces the potential error at a distance, the error for small values of *d *is weighted more heavily. We show that the error in *M *can be tightly bound using a grid-based sampling technique to determine points on *N*_1 _and *N*_2_.

**Figure 3:**
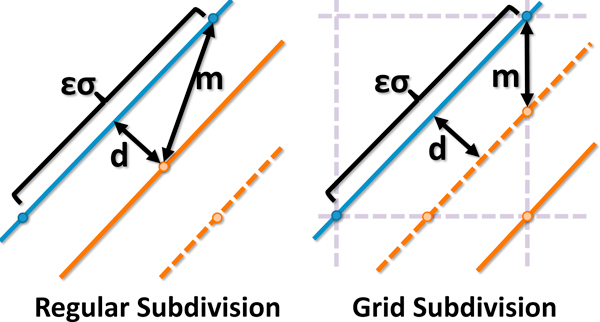
**Regular and Grid-based sampling methods where *d *is the actual distance and *m *is the measured distance**. (left) For regular subdivision, the worst-case error in the distance estimate $E\to \frac{\varepsilon \sigma }{2}$ as *d *→ 0. (right) Grid-based subdivision improves the worst case error while forcing *E *→ 0 as *d *→ 0. The difference in error becomes even more significant when scaled by the nonlinear metric function (Equation 3).

In the case of a regular sampling interval of *εσ *along all fibers, the maximum error in *M *is bounded by the function

(6) $$O\left(E\right)=1-e^{\frac{-\varepsilon^{2}}{8}}$$

for all *ε *> 0 (Figure 3). While this only occurs when *d*(*x, N*) ≈ 0, small distance values are generally desired when comparing networks.

We place a tighter error bound on *M *by placing sample points for both *N*_1 _and *N*_2 _on a common uniform grid (Figure 3). Sample points are selected at positions where a fiber crosses grid cell boundaries. Using a uniform grid with nodes of size *εσ*, the maximum error is

(7) $$O\left(E\right)=e^{\frac{-\varepsilon^{2}}{8}}-e^{\frac{-\varepsilon^{2}}{4}}$$

for all *ε *≤ 1. This upper error bound occurs at a distance of $\frac{\varepsilon \sigma }{2}$ (Figure 3) and the resulting error in *M *decreases as *d*(*x, N*) → 0. By using a value of $\varepsilon =\frac{1}{10}$, the largest error $E_{M}<\frac{1}{1000}$.

### Connectivity

**Figure 4:**
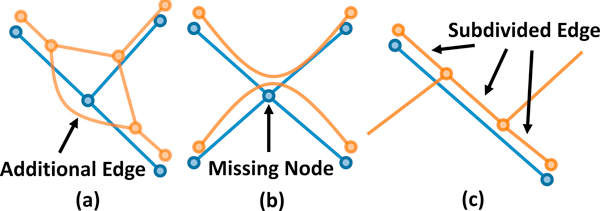
**Differences in topology can make network connectivity difficult to evaluate**. (a-b) A single missing node or additional edge can alter the connectivity connectivity. (c) Multiple edges in one graph can correspond to a single edge in the other.

Network connectivity is determined by converting each network to a graph based on the previous definitions. A network *N *is converted to a graph, *G *= {*V*,*E*} where *V *are nodes corresponding to fiber ends and intersections and *E *correspond to lengths of fiber connecting the corresponding nodes. Comparing network connectivity is therefore related to the graph isomorphism problem [30], for which there are no known polynomial-time solutions. Additional complexity is added since segmentation errors can introduce vertices and edges in one graph that do not exist in the other (Figure 4).

Because of this complexity, we inform our connectivity metric using key pieces of geometric information. In particular, our algorithm creates a mapping between detected nodes in the test-case to those in the ground-truth. In the ideal case where all nodes in the ground truth are detected, comparing connectivity is a trivial matter of finding edges that are present in both the ground-truth and test-case. However, this is insufficient when nodes are present in only one network. In particular, undetected or falsely detected nodes cause edges to become subdivided or result in topological changes (Figure 4). This removes the one-to-one correspondence of edges between the ground-truth and test-case. Our proposed metric estimates the number of undetected and falsely detected edges and vertices. In addition, we create a mapping between edge sequences in both networks, allowing interactive visualization of detected paths between nodes.

### Common connectivity errors

Common connectivity errors include additional edges and gaps. In the case of thinning algorithms, these edges are often due to high-frequency noise which form loops or spines on existing fibers. Many segmentation methods produce small gaps in fibers, forming multiple discontinuous segments in place of a single continuous fiber. These errors are difficult to detect geometrically, since disconnected points can occupy the same spatial position in the geometric model. The purpose of the proposed connectivity metric is to give equal weight to graph edges, independent of the length of the associated fibers.

### Connectivity metric

The proposed connectivity metric quantifies the quality of a segmentation based on the rate of false positives and false negatives, similar to the proposed method for geometry. The graphs *G_T _*= {*V_T_*,*E_T_*} and *G_GT _*= {*V_GT_*,*E_GT_*} are constructed using connectivity information in the test-case and ground-truth networks respectively. The geometric position of each node is then used to map between detected nodes *G_T _*and corresponding nodes in *G_GT_*. Undetected nodes are then eliminated to determine the *core connectivity*, describing the connectivity between detected nodes. These results are compared to find false-positive and false negative rates:

(8) $$C_{FNR}=\frac{FN}{FN+TP}$$

(9) $$C_{FPR}=\frac{FP}{FP+TP}$$

where *FN *is the number of edges in the ground-truth that are not represented in the test case, *FP *is the number of edges in the test-case that do not exist in the ground-truth, and *TP *is the total number of correctly detected edges.

#### Graph initialization

Each node is initialized with a three-dimensional coordinate from the explicit model. Each node *v *∈ *V_T _*∪ *V_GT _*is then assigned a *color *value based on the geometric positions of nodes in the ground-truth. The vertex *color *is a unique identifier that links nodes in both graphs that correspond to the same geometric feature in the original data set. A negative color value indicates that a node exists in one graph, but not in the other. To clarify, nodes that exist in both graphs are assigned a color value *C*(*v*) ≥ 0 and are referred to as *colored nodes*. Nodes that exist in only one graph are *uncolored *and have a color value of *C*(*v*) = -1.

All colors are initialized to *C*(*v*) = -1. For all *v_i _*∈ *V_GT_*, we find the closest node in *V_T_*:

(10) $$v_{j}=nearest\left(v_{i},V_{T}\right)$$

and assign a color to *v_i _*and *v_j_*:

(11) $$C\left(v_{i}\right)=C\left(v_{j}\right)=\left\{\begin{array}{cc} i & |v_{i}-v_{j}|<\sigma \\ -1 & \text{otherwise} \end{array}\right)$$

The color of each vertex is a unique identifier indicating the nearest node in *G_GT_*. A negative value is assigned if there are no nodes within a distance of *σ*. This color value provides the basis for comparing connectivity between the two networks.

All edges *e *∈ *E_T _*∪ *E_GT _*are initialized with a weight based on the geometry metric result for the associated fiber:

(12) W(e)=|e|M(e)

where |*e*| is the corresponding fiber length and *M*(*e*) is the geometry metric for the associated fiber. The value for *M*(*e*) is determined by integrating the geometry metric along a single fiber.

#### Core connectivity

We define the core connectivity of a graph *G *= {*V, E*} as the graph *G_c _*= {*V_c_, E_c_*}, where *V_c _*includes all nodes *v *∈ *V *where *C*(*v*) ≥ 0 and *E_c _*represents paths between elements of *V_c _*consisting of only uncolored nodes. Alternatively, given a graph *G *with colored nodes, we place an edge *e *= (*v_i_, v_j_*) in *G_c _*if there is a corresponding path in *G *between ${\hat{v}}_{i}$ and ${\hat{v}}_{j}$, where $C\left(v_{i}\right)=C\left({\hat{v}}_{i}\right)$ and $C\left(v_{j}\right)=C\left({\hat{v}}_{j}\right)$, that contains only uncolored nodes.

**Figure 5:**
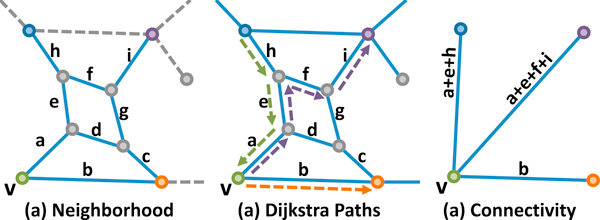
**Neighborhood search from a single node *v *(green) to its neighbors**. Color indicates *C*(*v_i_*) ≥ 0 and gray nodes indicate *C*(*v_i_*) = -1. (a) The local neighborhood includes all nodes reachable from *v*, bounded by nodes with a positive color value. (b) The resulting paths computed using a shortest-path search. (c) The core connectivity graph *G_c _*showing only edges incident on *v*.

**Figure 6:**
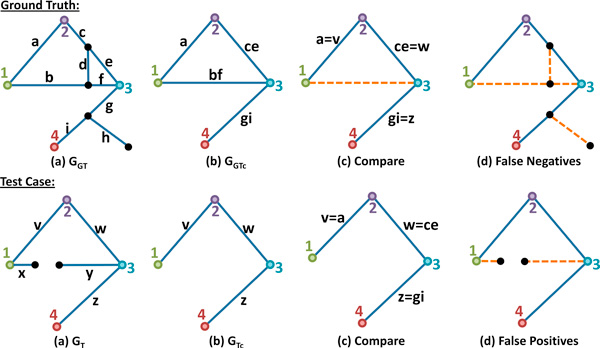
**Evaluating the core connectivity**. (a) Graph coloring uses a nearest-neighbor search to associate nodes in the test case to those in the ground truth. Nodes with color *C*(*v*) ≥ 0 are inserted into *G_c_*.(b-d) A breadth-first searche is used to find connected neighbors among the colored vertices. The edges forming the shortest path to each neighbor are combined to form a single graph in *G_c_*. The use of edge weights in the computation biases the selection of edges to those that have a high degree of geometric correspondence between the two networks.

Given a node *v *∈ *V *, all paths out of *v *are determined by using Dijkstra's shortest path algorithm [31] to explore the local neighborhood of *v*, bounded by nodes where *C*(*v_i_*) ≥ 0. This neighborhood is determined by performing a breadth-first search starting at *v*, where any branch of the search tree is terminated when it encounters a node *v_i _*where *C*(*v_i_*) ≥ 0 (Figure 5). Once the connectivity for a node is established, it is removed from *G *in order to prevent the insertion of redundant edges into *G_c _*(Figure 6).

#### Comparison

**Figure 7:**
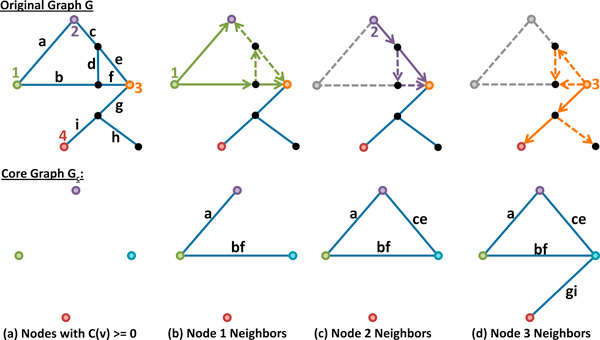
**Evaluating the connectivity metric**. (a) The initial graphs representing *N*_1 _and *N*_2 _are shown with colored nodes. (b) The core connectivity is computed by combining edges that produce the shortest paths to adjacent colored nodes. (c) The graphs representing core connectivity are then compared to find inconsistencies. (d) Valid connections are then mapped back to the original graphs to determine the false-positive rate and false-negative rate.

Once the core connectivity is established, the resulting core graphs *G_GTc _*and *G_Tc _*represent the connectivity between detected nodes in the ground-truth and test-case, respectively. These graphs are directly compared to find corresponding edges representing accurately detected connections. Edges in the original graphs that are members of these connections are true positives. Edges in *G_T _*that are not used in *G_Tc _*are false-positives and edges in *G_GT _*that are not used to form *G_GTc _*are false-negatives. The final value for FN is the sum of: (a) all vertices in *G_GT _*that have a negative color value and (b) all edges in *G_GT _*that are not used to build *G_GTc_*. The value for FP is determined in the same way for the test-case. An overview of the algorithm for the connectivity metric is shown in Figure 7.

### Implementation

Graph coloring is performed using a nearest-neighbor search, similar to the method described in Section . This search requires a maximum *O*(*N *log *N*) time, where *N *is the number of nodes in the largest graph. Computing the local neighborhood is incorporated into the minimum path algorithm by ending the current search iteration when a node with *C*(*v*) ≥ 0 is discovered. Therefore, no paths passing through a colored node are considered.

The shortest-path algorithm has a time complexity of $O\left(\hat{E}+\hat{V}\text{log}\hat{V}\right)$[32], where $\hat{E}$ and $\hat{V}$ are the number of edges and nodes in the local neighborhood. This search must be computed for every node in a graph, resulting in a time complexity of $O\left(N\hat{V}\text{log}\hat{V}\right)$. Biological networks are believed to be scale-free [33], therefore the number of edges per node is expected to be small. A poor segmentation can produce a network with a significant number of nodes, resulting in a time-consuming analysis, therefore this complexity must be considered. In practice, however, the local neighborhood tends to be small. For these cases, $\hat{V}\text{log}\hat{V}$ can be considered constant.

### Visualization

The geometry and connectivity metrics proposed in this paper provide a global measure for comparing interconnected networks. However, one of the principle advantages of the proposed algorithm is the ability to localize geometric and connectivity errors. If properly visualized, this can allow developers to quickly identify cases where segmentation algorithms fail and provide insight into improving algorithms. In this section, we describe techniques that we have employed to visualize the differences between networks.

Several methods have been proposed for visualizing fiber structures, particularly in the field of diffusion tensor MRI. The most common methods use streamlines and stream tubes [34]. Methods for visualizing networks by applying orientation filters [35] have been proposed. In addition, selective visualization of volumetric data [36] has been used to render networks with similar structure to those described in this paper. The rendering methods that we use are inspired by recent techniques for rendering high-dimensional functions using three-dimensional connected lattices [37].

### Color mapping

**Figure 8:**
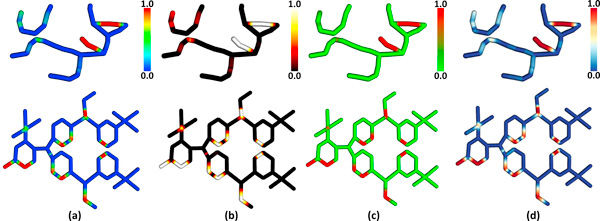
**Colormapping for visualizing geometric error**. (a) Rainbow colormapping is frequently used to characterize scalar fields and can make prominent errors, such as unsegmented filaments, easy to identify. (b) Blackbody radiation has been shown to provide a better perceptual indication of varying scalar fields, however this mapping often obscures shading, which provides context for three-dimensional structure. (c) Isoluminant shading overcomes these problems at the expense of lower dynamic range. (d) A diverging color (default) maps from cool to warm hues, providing higher dynamic range without obscuring shading.

The selection of appropriate color maps for scalar field visualization is a difficult problem, particularly when the scalar field is mapped onto a three-dimensional structure. The rainbow color map (Figure 8a) is frequently used because it provides a high dynamic range of color values by varying hue as a function of the scalar field. However, previous work has shown that that rainbow color maps provide misleading results compared to isoluminant and blackbody color maps [38]. One reason for this is that changes in hue are not interpreted uniformly by the human visual system, resulting in (a) the supression of subtle changes in a scalar field and (b) the introduction of artifacts due perceived bands in the color map, which is often interpreted as an artificial segmentation of the data. Alternative approaches, such as blackbody radiation color maps (Figure 8b) and isoluminant color maps (Figure 8c), eliminate some of these problems. However, isoluminant color maps provide lower dynamic range, since only two colors are interpolated, and cannot be interpreted by the large number of people (≈30%) who exhibit color blindness. The use of blackbody radiation color mapping provides a higher dynamic range, however the change in illumination intensity interferes with surface shading, which provides important cues as to the shape of a three-dimensional structure. When mapped onto our proposed network representation, this can make understanding the three-dimensional structure of the network difficult.

Divergent color mapping (Figure 8d) provides an intuitive ordering from cool to warm colors and can be used with shading, making it useful for surface-mapping on three-dimensional models. In addition, recent work by Borkin et al. [39] has shown that diverging color schemes significantly improve the interpretation of scalar data on tube-like structures when compared to rainbow color mapping. The NetMets software supports all four color mapping methods, with the blue-red divergent color scheme as the default.

### Geometry

We visualize geometric differences by mapping the geometry metric directly onto the explicit representation of the original models. The global geometry metric described earlier is evaluated by integrating along the curves representing fibers in the network model *N*_1_. The resulting value provides a very general measure of the average distance between *N*_1 _and another network *N*_2_. For visualization, the value at each point on *N*_1 _is used to highlight the specific differences in geometry between *N*_1 _and *N*_2_.

**Figure 9:**
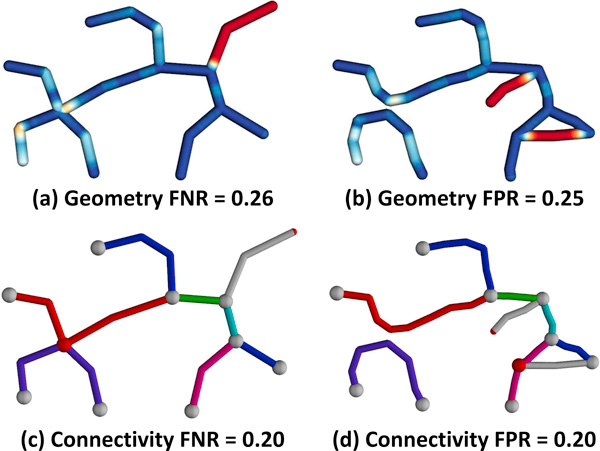
**Hierarchical proxy network comparison**. Geometric error for the (a) ground-truth and (b) test-case are shown. Hue indicates the value of the geometry metric, where blue indicates a strong correspondence and red indicates an error. (c-d) Connectivity shows mapped edges rendered in the same color. Undetected nodes are rendered in red and unmapped edges are white.

**Figure 10:**
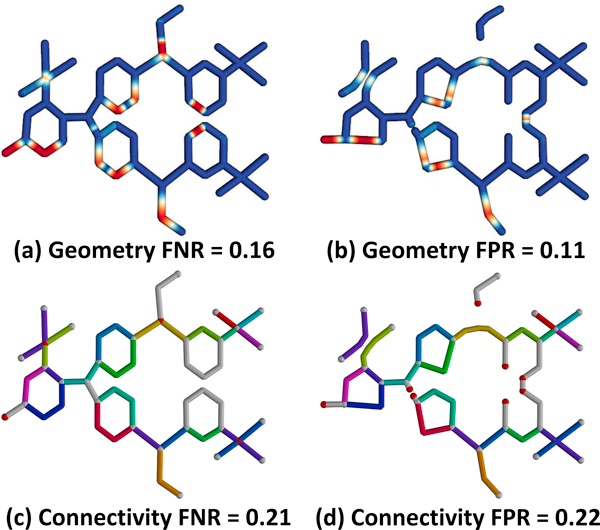
**Interconnected network representing an organic molecule**. Geometric error for the (a) ground-truth and (b) test-case are shown, where red indicates significant deviation. (c-d) Connectivity shows mapped edges rendered in the same color. Undetected nodes are rendered in red and unmapped edges are white.

We display this information by extruding a tube along all fibers in *N*_1_. A colormap is applied to indicate the value of the weighted *N*_2 _distance field (Equation 3) at each point on *N*_1_. This is implemented by storing the value of the weighted distance field at each point in the explicit model:

(13) $$M_{N_{1}}\left(x\right)=1-e^{\frac{d{\left(x,N_{2}\right)}^{2}}{2\sigma^{2}}}$$

where *x *is a vertex on the explicit model. These values are stored with the points that make up the geometry in the explicit model. The data for the metric is displayed by passing the value of *M_N_*(*x*) to the GPU as a texture coordinate along with the vertex position *x*. A fragment shader converts the value to the appropriate color. This is demonstrated for both hierarchical (Figure 9) and interconnected (Figure 10) proxy models. As seen in the figures, red regions on the model indicate errors in segmentation. In the case of the ground-truth, these fibers were undetected. In the case of the test-case, red fibers are falsely detected.

### Connectivity

The concept of network connectivity is significantly more abstract, since a one-to-one correspondence between fibers in *N*_1 _and *N*_2 _often does not exist. For example, fibers that are subdivided (Figure 4) can result in a single fiber in *N*_1 _being mapped to multiple fibers in *N*_2_. This can also result in multiple fibers in *N*_1 _overlapping when mapped to *N*_2_. In the case of spurious or undetected fibers, a mapping between *N*_1 _and *N*_2 _does not exist.

We use several methods to visualize errors in connectivity. First of all, undetected or spurious nodes are rendered as red spheres, while detected nodes are gray. This simple strategy is used to visualize regions where connectivity errors are frequently made. Where a mapping exists between edges in the ground truth and test case, corresponding edges are color-coded. This is shown for both hierarchical (Figure 9) and interconnected (Figure 10) proxy data. The mapping is based on common edges found in the core connectivity graphs for each network (Figure 6). Edges that did not exist in the core connectivity graph, or were later removed, are rendered in gray.

**Figure 11:**
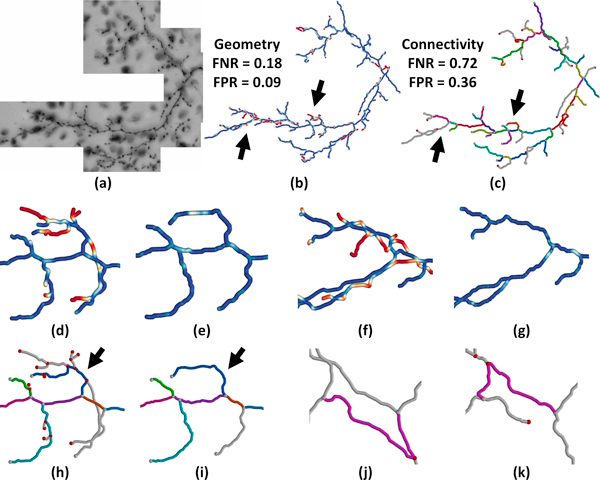
**Segmentation of cerebellar climbing fibers from the DIADEM Challenge data set **[17]. (a) Minimum intensity projection of the raw image data set. (b) The ground-truth model with geometric error rendered using a diverging color map. (c) The test-case model shows detected edges rendered in different colors. Undetected edges are gray. (d-g) A close-up of the indicated regions (arrows) showing geometric error for the ground-truth (left) and test cases (right). (h-i) Edge mapping between the ground-truth and test case. Undetected nodes are shown in red. (j-k) Edge-mapping provides an estimate of fiber correspondence in regions that are incorrectly connected. The result of the DIADEM Metric for this data set is *M *= 0.315.

Finally, allowing the selection of fibers is an important feature for understanding errors that can occur in connectivity. This is seen in the visualization of cerebellar fibers from the DIADEM data set (Figure 11g and 11h). In this case, a local error in connectivity can cause significant changes when the model is considered hierarchically. In this case, an edge mapping was found between the two models. Selectively visualizing this edge makes the nature of the error easier to understand.

## Results

In this section, we demonstrate how the NetMets software can be used to compare explicit interconnected networks in several cases relevant to current research needs. We first show how NetMets can be used evaluate the performance of an automated segmentation algorithm on a data set distributed as part of the DIADEM Challenge. We then evaluate the performance of the same algorithm on fluorescence microscopy data. Next, we show that NetMets can be useful for comparing different manual tracings of the same network structure. Finally, we demonstrate how the bi-directional measurement used by our proposed metric algorithm can be useful in evaluating segmentation effectiveness when only an incomplete ground truth is available.

### Evaluating segmentation algorithms

One of the primary motivations for this work is to provide a quantitative method for evaluating the performance of segmentation algorithms as well as an intuitive visualization approach for identifying where segmentation errors arise in the data. We show how NetMets is suited for this task by performing automated segmentation of two data sets. The first data set is a bright-field microscopy image of a series of cerebellar climbing fibers. This data set is distributed through the DIADEM Challenge [40] (Dataset 1) and is available online http://www.diademchallenge.org. The model used as the ground truth was created for the DIADEM Challenge and is distributed with the data. The second data set is mouse brain tissue imaged using a confocal microscope. The data set contains a network of astrocytes in the neighborhood of a blood vessel. The ground truth was manually constructed using Neuromantic http://www.reading.ac.uk/neuromantic/.

Automated segmentation was performed using ridge detection, followed by dilation and topology-preserving thinning [5] to produce an implicit skeleton. Voxels composing the skeleton were then explicitly connected and smoothed to perform the final model. While superior algorithms have been described [41-45], a basic algorithm is useful in this case since our goal is to demonstrate how errors can be quantified and localized.

Segmentation of the cerebellar climbing fibers data set produces a reasonable model of the prominent geometric features (Figure 11). The results of the metric indicate that approximately 18% of the ground truth geometry is missed by the segmentation while approximately 72% of the ground truth connections are undetected. Close inspection shows that missed regions correspond to short fibers (Figure 11d) or fibers with a close proximity to detected fibers (Figure 11f). The large number for false-negative connectivity corresponds to the significant number of small fibers missed by the automated algorithm (Figure 11h). Since this data set is directly supported by the DIADEM Metric (*D *= 1) and the resulting model is tree-like, we can evaluate the DIADEM Metric as *M *= 0.315. For our comparison, we use a standard deviation of *σ *= 10 pixels. Note the additional information provided by NetMets and the ability to visualize the errors on the network models.

**Figure 12:**
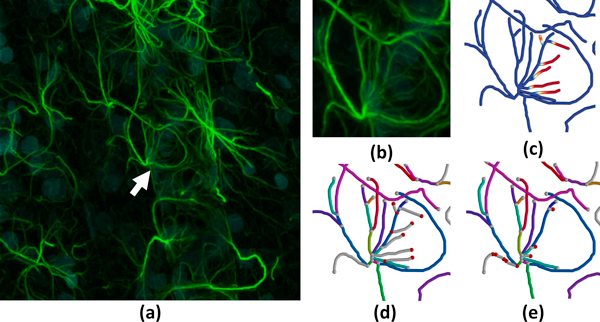
**Validation of an automated segmentation algorithm on an astrocyte network**. (a) A maximum intensity projection of the image stack with (b) close-up. (c) Mapping of the geometric error onto the ground-truth model. The edge mapping and connectivity is shown for both the (d) ground truth and (e) test case.

Our second data set consists of a stack of confocal images of an astrocyte network in close proximity to a blood vessel in the mouse brain. We apply the same automated skeletonization algorithm to these images after inverting their intensity. Upon close inspection of the resulting model we find errors similar to those in our previous data set, where small and low-intensity fibers are undetected (Figure 12). Also, note that the ground truth and test models consist of multiple trees which the proposed algorithm can handle robustly.

### Comparing manual segmentations

While a significant amount of current research in the area of neuronal segmentation is directed toward fully-automated reconstruction, building an accurate ground-truth can also be a difficult problem. This is particularly true for extremely dense and complex data sets, where manual segmentation is a time-consuming process that introduces fatigue in the experts producing the desired model. Recent work by Helmstaedter et al. [46] demonstrate a method for overcoming this problem by using multiple experts to trace a single data set. The models for each expert can then be used to produce a more accurate ground-truth.

**Figure 13:**
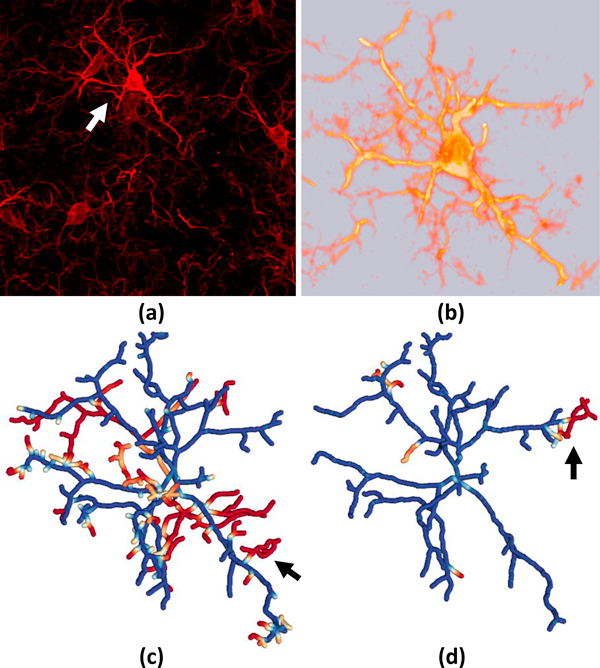
**Comparing two manually-constructed models of microglia**. Both models were traced using Neuromantic by human operators. (a) A maximum intensity projection of the original confocal image stack and (b) a volume visualization of the target microglia. (c-d) The geometric error is shown on both models.

In this section, we demonstrate the use of NetMets for comparing the results of two manually-constructed models from a confocal image stack of mouse brain microglia (Figure 13). A single cell was selected for segmentation. Both models were created using Neuromantic and consist of a single tree-like network with a matching root node at the soma. Visualization of the data in NetMets indicates that the primary differences between the models are the low-contrast fibers in deep sections of the data set where there is more ambiguity over which processes belong to the selected soma. Since our algorithm allows these differences to be localized, users can explore individual segments that exhibit a large amount of error and focus on resolving those ambiguities. This could potentially allow more efficient use of time among experts by allowing them to focus on ambiguous fibers rather than independently tracing the entire model.

### Subgraph comparison

With the development of new high-throughput imaging methods, the size and complexity of data sets can make it impractical to construct a complete ground truth. One possible solution to this problem is to manually label small subsets of the raw data. However, thorough validation of a large data set would require manual labeling of several small subsets to provide a statistically viable sample size. This often makes manual tracing more complex by introducing artificial fiber terminations at the boundaries of these subsets. Since current tools like Neuromantic allow semiautomated tracing, it is often easier to manually trace long fibers than to start and terminate several small ones. It would therefore be convenient to create a ground truth that represents a subset of complete fibers in the data set. However, this would cause properly segmented fibers to be incorrectly labeled as false-positives when there is no corresponding segmentation in the ground-truth. One of the advantages of using a bi-directional measurement like the one we have proposed is that this case can be, to some extent, recognized and corrected.

**Figure 14:**
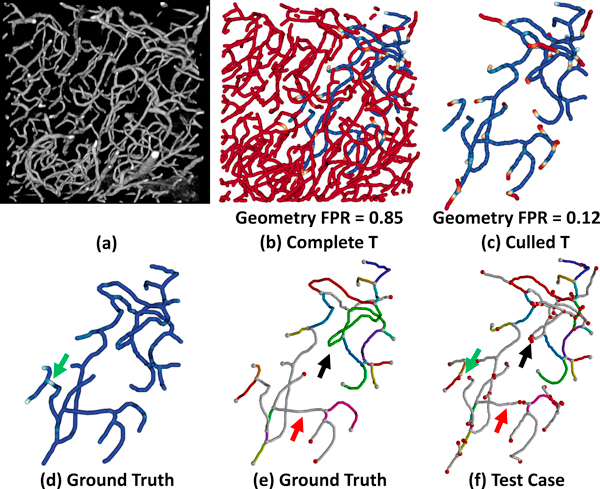
**Evaluating a segmentation with an incomplete ground truth**. (a) Volume visualization of raw KESM data showing microvessels in the mouse brain. (b) The complete test case compared to an incomplete ground truth. Red fibers indicate tracked vessels not present in the ground-truth model. (c) Fibers culled by setting a geometric error threshold of 0.9. (d) Geometric error in the ground-truth model. The connectivity graph is shown for the (e) ground-truth and (f) test-case. Red and green arrows indicate breaks in the test-case fibers, resulting in connectivity errors. Black arrow indicates an incorrectly mapped edge.

We demonstrate this by creating an incomplete ground truth for a mouse brain microvascular data set imaged using a high-throughput imaging technique called Knife-Edge Scanning Microscopy (KESM) [47]. The data set is then segmented using a topology-preserving thinning algorithm. Given the high contrast of the data, curvelet preprocessing is not necessary. A volume visualization of the data set and the NetMets comparison are shown in Figure 14. The geometric false negative rate is low (FNR = 0.05), indicating that very few fibers are missed by the automated skeletonization. However, the false positive rate is extremely high (FPR = 0.85). In order to gain a better understanding of the effectiveness of the tracking algorithm, we cull all fibers from the test case that have a mean error value greater than 0.9. This allows us to gain a better understanding of how the algorithm is behaving on a global scale while allowing us to examine connectivity errors that occur in detected fibers. Performing this culling results in a FPR of 0.12.

## Conclusion and future work

In this paper, we propose robust methods for quantifying and visualizing differences in interconnected fiber networks. This work is motivated by the need to validate segmentation algorithms for interconnected networks in biomedical imaging. As biomedical data sets increase in size and complexity, qualitative comparison has become insufficient to address this issue. The techniques that we propose build on the quantification principles introduced by the DIADEM Challenge [16] and serve as a basis for building visualization tools that extend both quantitative and qualitative validation to research on large-scale biological networks.

Current advances in high-throughput imaging are motivating research into robust and generalized segmentation algorithms, which are particularly useful in the field of connectomics [26]. Opportunities exist for finding common segmentation errors across a large network. In particular, correlating connectivity and geometric errors with features in the original data set could help train segmentation algorithms. In addition, our algorithm requires a qualified ground-truth in order to perform the comparison, which is difficult to create for large data sets. We demonstrate that subsets of the ground truth can be used to estimate the effectiveness of a segmentation algorithm, however this is based on culling high-error fibers from the test case. This can result in the exclusion of fibers that are inaccurately segmented, resulting in overestimation of the algorithm's performance. This is something that may be addressed with more complex culling algorithms based on other fiber features such as length and connectivity.

In addition, more accurate edge mapping between the ground truth and test cases would be useful for visualization, since one of the more common errors we have found in our tracing examples are fibers that terminate early, putting them out of range of the corresponding ground truth end node. While this is taken into account in the geometry metric, it can provide for confusing visualization when exploring the connectivity graph.

Finally, previous methods such as the DIADEM Metric provide advantages that may improve the effectiveness of our proposed algorithms. In particular, the use of fiber length as a geometric measurement can capture errors that are not recognized by our algorithm, such as erroneous fibers that are in close proximity to actual geometry. The NetMets software is available online as open source at http://www.davidmayerich.net/software.

## List of abbreviations used

MSE: Mean Squared Error; DIADEM: Digital Reconstruction of Axonal and Dendritic Morphology; FPR: False Positive Rate; FNR: False Negative Rate; TED: Tree Edit Distance; FN: False Negative; FP: False Positive; KESM: Knife-Edge Scanning Microscopy.

## Competing interests

The authors declare that they have no competing interests.

## Authors' contributions

D. Mayerich implemented the NetMets software and imaged KESM data, C. Bjornsson prepared and imaged the tissue samples and created ground-truth models, J. Taylor created ground-truth models and performed automated segmentation, B. Roysam developed automated segmentation algorithms and metrics.
